# The Coordination and Luminescence of the Eu(III) Complexes with the Polymers (PMMA, PVP)

**DOI:** 10.3390/polym10050508

**Published:** 2018-05-07

**Authors:** Weican Zhao, Haifeng Shao, Guang Yu, Yanjun Hou, Shuhong Wang

**Affiliations:** 1 School of Chemistry and Materials Science, Heilongjiang University, Harbin 150086, China; 2151165@s.hlju.edu.cn (W.Z.); 2141100@s.hlju.edu.cn (H.S.); 2141097@s.hlju.edu.cn (G.Y.); 2 Key Laboratory of Functional Inorganic Material Chemistry, Heilongjiang University, Harbin 150086, China

**Keywords:** complexes, luminescent, PMMA, composite nanofibers

## Abstract

The rare earth complexes and the polymers can be made into composite nanofibers through electrospinning. The fluorescence intensity of these fiber composites is much higher than that of the rare earth complexes. By changing the mixed proportion of polymethyl methacrylate (PMMA) and complexes, nanofiber materials were prepared. Then, by measuring their fluorescence intensity, it is found that the carbonyl bond of PMMA may have coordinated with the rare earth ions and enhanced the luminescence intensity of them. Then, a series of experiments were designed to study their coordination and luminescence mechanism. The coordination mechanism of the polymers with carbonyl groups and the rare earth complexes was explained by Eu(TFT)_3_(TPPO), Eu(TFT)_3_(TPPO)_2_, Eu(PFP)_3_(TPPO), Eu(PFP)_3_(TPPO)_2_, and polyvinyl pyrrolidone (PVP) dissolved in chloroform solution, where TFT means 2-(2,2,2-trifluoroethyl)-1-tetralone, PFP means 2-(2,2,3,3,3-Pentafluoropro-panoyl)-3,4-dihydronaphthalen-1(2H)-one and TPPO means phosphine oxide. The coordination of PVP and the rare earth complexes in solution was studied, and it was found that the fluorine atoms of the ligand had a significant impact on the aggregation-induced effect of the composites. The electron transitioned in the polymers and the complexes were enhanced greatly by the coordination. The colors of emission light could be adjusted by the coordination of the polymers and the rare earth complexes.

## 1. Introduction

Lanthanide ions are widely used in the field of optical and magnetic researches on the basis of their peculiar luminescent and magnetic properties [1,2,3,4,5,6]. However, Ln(III) ions are unable to show a high luminous emission under direct excitation because the f–f transitions of lanthanide ions are spin-forbidden. Many scientists find that the auxiliary ligands can play an important role in increasing the intensity of a luminous emission and inhibiting the quenching of fluorescence by an intramolecular energy transfer process from ancillary ligands to the central metal ion—the so-called ‘‘synergistic effect’’ [7]. The design of lanthanide coordination compounds has attracted particular attention in order to improve the luminescent properties of lanthanide ions.

In recent years, people with knowledge of the rare earth complexes attempted to take advantage of the polymers, which can be used as ligands to synthesize a series of macromolecular complexes. These macromolecular complexes have many unique properties [8,9,10,11,12,13]. Some polymers with carbonyl groups have been mixed with the rare earth complexes to be made into nanospinning materials. The fluorescence properties of nanospinning materials were studied, and their properties have been improved greatly [14,15]. However, the in-depth studies of their mechanism have not been reported. We all know that the molecular electron transitions of macromolecular polymers are different to those of the rare earth complexes [16,17].

In our previous study, 2-(2,2,2-trifluoroethyl)-1-tetralone (TFT) and 2-(2,2,3,3,3-Pentafluoropro-panoyl)-3,4-dihydronaphthalen-1(2H)-one (PFP) were used to synthesize firm coordination compounds with Eu^3+^ ions in order to enhance the luminescence intensity due to the effect of their π–π* transition [18,19,20,21,22,23]. In this paper, we further synthesized two ligands and six complexes. The coordination and luminescence mechanism of the complexes and the polymers were studied in different ways. The Eu(TFT)_3_phen/PMMA and Eu(TFT)_3_bpy/PMMA nanofibers were prepared by electrospinning technology, and the influence of Eu(III) complexes on both the morphology and the luminescence of composite nanofibers was researched. The synergistic luminescence mechanism of the complexes and the polymers was studied by the combination of some selected complexes with PVP. The polymers have a very strong characteristic emission. The rare earth complexes [24,25,26,27,28,29,30] and the polymers (carbonyl groups) can form unstable coordination, thus changing the fluorescence properties of the Eu(III) complexes [31,32,33,34]. Usually, the emitting colors from the electron transitions in the polymer molecules are blue, while the Eu(III) complexes emit red light. The emitting colors can be adjusted by the coordination of the polymers and the rare earth complexes.

## 2. Synthesis and Characterization

### 2.1. Synthesis of 2-(2,2,2-trifluoroethyl)-1-tetralone (TFT) and 2-(2,2,3,3,3-pentafluoropropanoyl)-3,4-dihydronaphthalen-1(2H)-one (PFP)

**Scheme 1:**
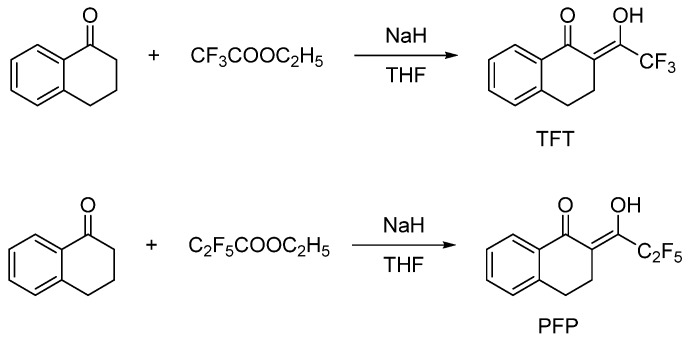
Structures of compounds 2-(2,2,2-trifluoroethyl)-1-tetralone (TFT) and 2-(2,2,3,3,3-Pentafluoropro-panoyl)-3,4-dihydronaphthalen-1(2H)-one (PFP).

1-tetralone (5.00 mmol) in THF (10 mL) was added to a THF solution (50 mL) of ethyl trifluoroacetate (ethyl pentafluoro-propionate) (10.00 mmol). The mixture was stirred for 1 h. Later, NaH (7.5 mmol) solids were added with vigorous stirring, and the combined solution was stirred at 25 °C for 24 h in an inert atmosphere. Then, a dilute hydrochloric acid solution (0.5 mol·L^−1^) was added to the solution to make the solution have a pH 2. The mixed solution was extracted twice by dichloromethane. The organic layer was separated from the aqueous layer and dried overnight with anhydrous Na_2_SO_4_. After the solvent was removed by reduced pressure distillation, an oily solid was obtained. The solid product was isolated by column chromatography using hexane as an eluent (Scheme 1).

TFT: Yellow crystal. Yield: 81%. m.p. 50.2–50.8 °C. ^1^H NMR (400 MHz, CDCl_3_) δ 15.66 (s, 1H), 7.99 (d, *J* = 7.8 Hz, 1H), 7.50 (td, *J* = 7.5, 1.4 Hz, 1H), 7.37 (t, *J* = 7.4 Hz, 1H), 7.28–7.25 (m, 1H), 3.49 (s, 1H), 2.94–2.87 (m, 1H), 2.77 (t, *J* = 6.8 Hz, 1H) IR (KBr) νmax: 2947 cm^−1^ (s, ν_O–H_), 1600 cm^−1^ (s, ν_C=O_), 1303 cm^−1^ (s), 1246 cm^−1^ (s), 1139 cm^−1^ (s, ν_C–F_), 753 cm^−1^ (m, ν_CF3_).

PFP: Yellow solid. Yield: 79%. m.p. 57.4–57.9 °C. ^1^H NMR (400 MHz, CDCl_3_) δ 16.18 (s, 1H), 8.01 (d, *J* = 8.3 Hz, 1H), 7.52 (t, *J* = 7.5, 1H), 7.38 (t, *J* = 7.5 Hz, 1H), 7.27 (d, *J* = 6.1 Hz, 1H), 3.49 (s, 1H), 2.90 (dd, *J* = 8.7, 5.4 Hz, 2H), 2.84–2.78 (m, 2H). IR (KBr) νmax: 2924 cm^−1^ (s, ν_O–H_), 1597 cm^−1^ (s, ν_C=O_), 1324 cm^−1^ (s), 1222cm^−1^ (s), 1158 cm^−1^ (s, ν_C–F_), 742 cm^−1^ (m, ν_CF3_).

**Figure A1:**
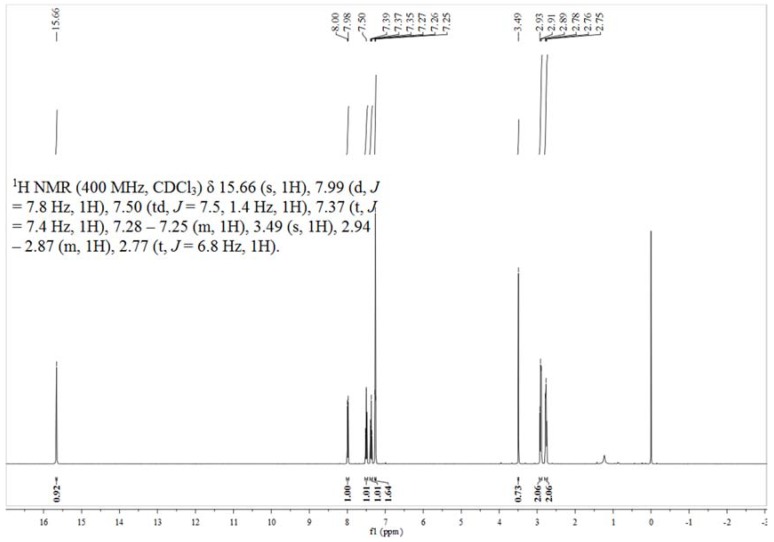
^1^H NMR spectra of TFT.

**Figure A2:**
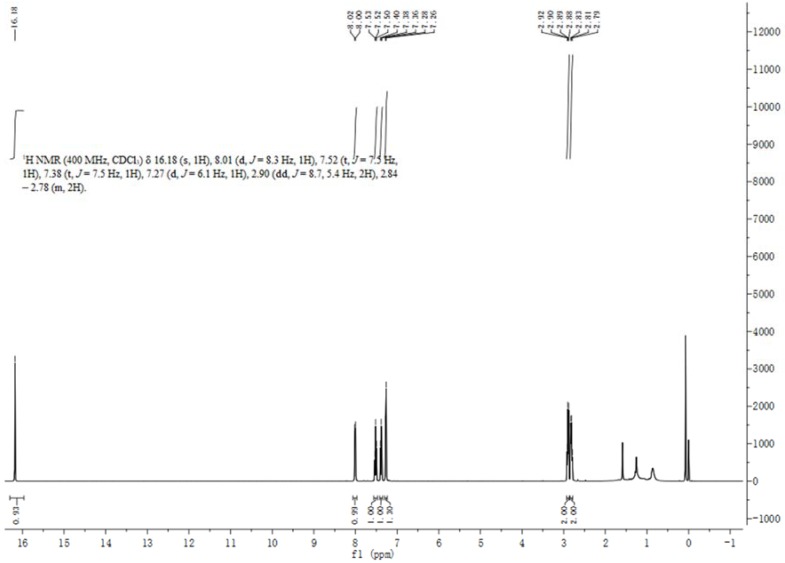
^1^H NMR spectra of PFP.

The ^1^H NMR spectra of TFT and PFP are shown in Appendix A (Figure A1 and Figure A2).

### 2.2. Syntheses of Complexes ***1**–**6***

**Scheme 2:**
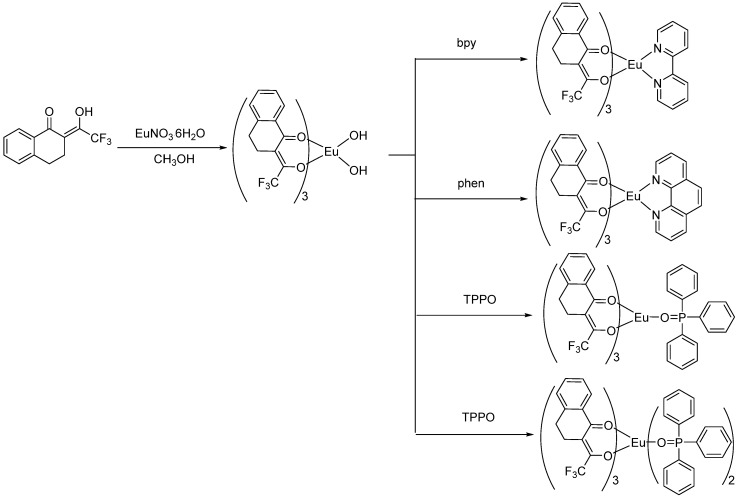
Syntheses of complexes **1**–**4**.

**Scheme 3:**
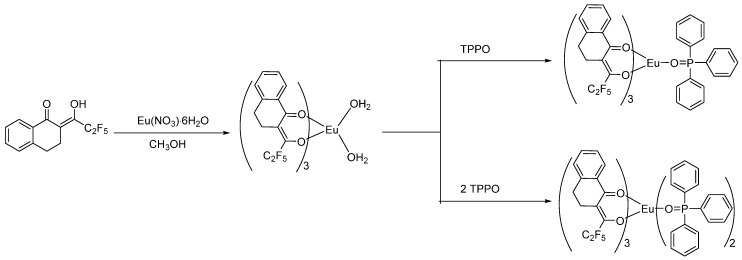
Syntheses of complexes **5** and **6**.

**Scheme 4:**
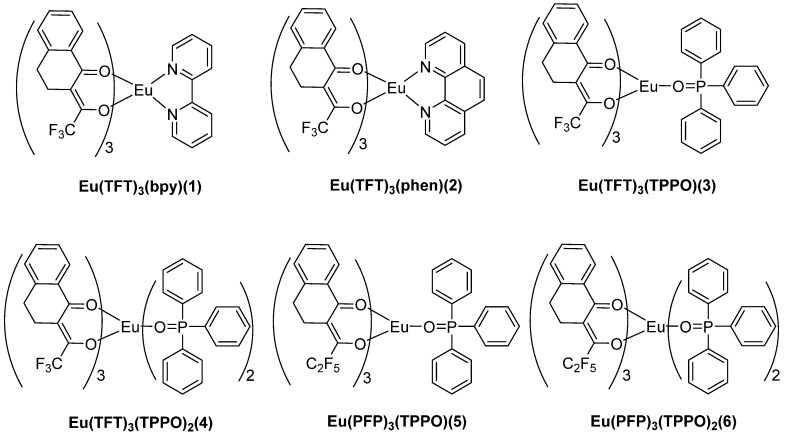
Structures of complexes **1**–**6**.

Mixed TFT (PFP) (4.12 mmol) and NaOH (4.12 mmol) in CH_3_OH (20 mL) was stirred for 0.5 h. Another methanol solution of EuCl_3_·6H_2_O (1.38 mmol) was added dropwise under stirring. Then, the substances 1,10-*o*-Phenanthroline (Phen) or 2,2-Bispyridine (Bpy), phosphine oxide (TPPO), and TPPO (2.76 mmol) (1.38 mmol) were added and stirred for 12 h at 25 °C (Scheme 2 and Scheme 3). The products were obtained by reduced pressure distillation and washed with hexane, then recrystallized with the dichloromethane-hexane (Scheme 2, Scheme 3 and Scheme 4).

Eu(TFT)_3_(bpy)(**1**): Yield: 82% Anal. (%) Calcd for C_46_H_32_EuN_2_O_6_F_9_ (*M*_r_ = 1031): C 53.54, H 3.10; Found: C 53.43, H 3.12. IR data (KBr pellet, cm^−1^): 1596 cm^−1^ (s, ν_C=O_), 1302 cm^−1^ (s), 1231 cm^−1^ (s), 1123 cm^−1^ (s, ν_C–F_), 754 cm^−1^ (m, ν_CF3_).

Eu(TFT)_3_(phen)(**2**): Yield: 87% Anal. (%) Calcd for C_48_H_32_EuN_2_O_6_F_9_ (*M*_r_ = 1055): C 54.60, H 3.05; Found: C 54.62, H 3.08. IR data (KBr pellet, cm^−1^): 1596 cm^−1^ (s, ν_C=O_), 1303 cm^−1^ (s), 1233 cm^−1^ (s), 1126 cm^−1^ (s, ν_C–F_), 755 cm^−1^ (m, ν_CF3_).

Eu(TFT)_3_(TPPO)(**3**): Yield: 79% Anal. (%) Calcd for C_54_H_39_EuPO_7_F_9_ (*M*_r_ = 1153): C 56.20, H 3.38; Found: C 56.18, H 3.35. IR data (KBr pellet, cm^−1^): 1595 cm^−1^ (s, ν_C=O_), 1302 cm^−1^ (s), 1234 cm^−1^ (s), 1120 cm^−1^ (s, ν_C–F_), 755 cm^−1^ (m, ν_CF3_).

Eu(TFT)_3_(TPPO)_2_(**4**): Yield: 82% Anal. (%) Calcd for C_72_H_54_EuP_2_O_8_F_9_ (*M*_r_ = 1431): C 60.38, H 3.77; Found: C 60.35, H 3.79. IR data (KBr pellet, cm^−1^): 1597 cm^−1^ (s, ν_C=O_), 1303 cm^−1^ (s), 1232 cm^−1^ (s), 1121 cm^−1^ (s, ν_C–F_), 754 cm^−1^ (m, ν_CF3_).

Eu(PFP)_3_(TPPO)(**5**): Yield: 82% Anal. (%) Calcd for C_57_H_39_EuPO_7_F_15_ (*M*_r_ = 1272): C 53.77, H 3.07; Found: C 53.74, H 3.12. IR data (KBr pellet, cm^−1^): 1595 cm^−1^ (s, ν_C=O_), 1291 cm^−1^ (s), 1221 cm^−1^ (s), 1124 cm^−1^ (s, ν_C–F_), 752 cm^−1^ (m, ν_CF3_).

Eu(PFP)_3_(TPPO)_2_(**6**): Yield: 83% Anal. (%) Calcd for C_75_H_54_EuP_2_O_8_F_15_ (*M*_r_ = 1581): C 56.93, H 3.42; Found: C 56.89, H 3.37. IR data (KBr pellet, cm^−1^): 1597 cm^−1^(s, ν_C=O_), 1291 cm^−1^ (s), 1227 cm^−1^ (s), 1122 cm^−1^ (s, ν_C–F_), 751 cm^−1^ (m, ν_CF3_).

The FTIR spectra of TFT, PFP, complexes **1**–**6** are shown in Appendix B (Figure A3, Figure A4, Figure A5, Figure A6, Figure A7, Figure A8, Figure A9, Figure A10, Figure A11 and Figure A12). FTIR spectra of TFT, complexes **2**–**4** and Eu(TFT)_3_(phen)/polymethyl methacrylate (PMMA), which was synthesized in subsequent experiments, are shown in Figure 1 for comparison purposes.

### 2.3. Molecular Structures of the Complexes **1** and **2**

**Figure 2:**
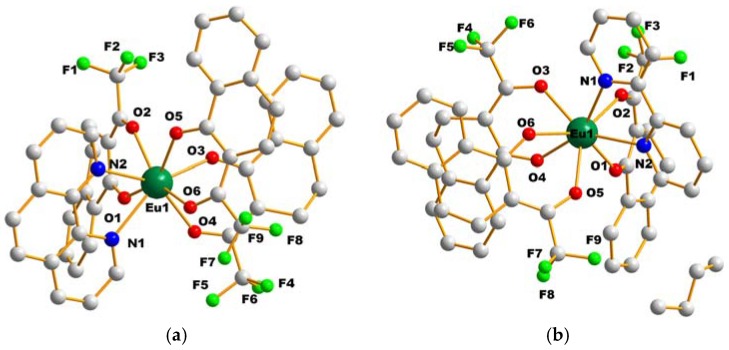
Molecular structure for Eu(TFT)_3_(phen) (**a**) and Eu(TFT)_3_ (bpy)·1/2Hex (**b**).

The molecular structures of the complexes **1** and **2** were revealed by single crystal X-ray studies, which crystallize in the monoclinic crystal system with space groups of *P*2_1_/*n* and *P*2_1_/*c*, respectively. The asymmetric unit of complex **1** is composed of one Eu^3+^ ion, three deprotonated ligands, and one auxiliary ligand (phen, bpy). Each Eu^3+^ ion center adopts a distorted hexahedron geometry, coordinated by six O atoms from three carbonyl groups (O1, O3, O5), three hydroxy groups (O2, O4, O6) of three ligands, and two N atoms (N1, N2) from one auxiliary ligand (Figure 2a,b). The average bond lengths of the Eu–O and Eu–N for complex **1** are 2.347 Å and 2.588 Å, respectively. The average bond lengths of the Eu–O and Eu–N for complex **2** are 2.357 Å and 2.583 Å (Appendix D, Table A1 and Table A2), respectively. The average length of the Eu–N bond with the nitrogen atoms from the auxiliary ligand is substantially longer, indicating a weak interaction. In other words, the TFT ligand is more stable when bound to the central metal ion, which is due to the strong interaction between the Eu^3+^ ion and the oxygen atoms. In comparison with complex **1**, complex **2** introduces the solvent n-hexane.

### 2.4. Morphology of Composite Nanofibers and Dispersion of Eu/PMMA.

**Figure 3:**
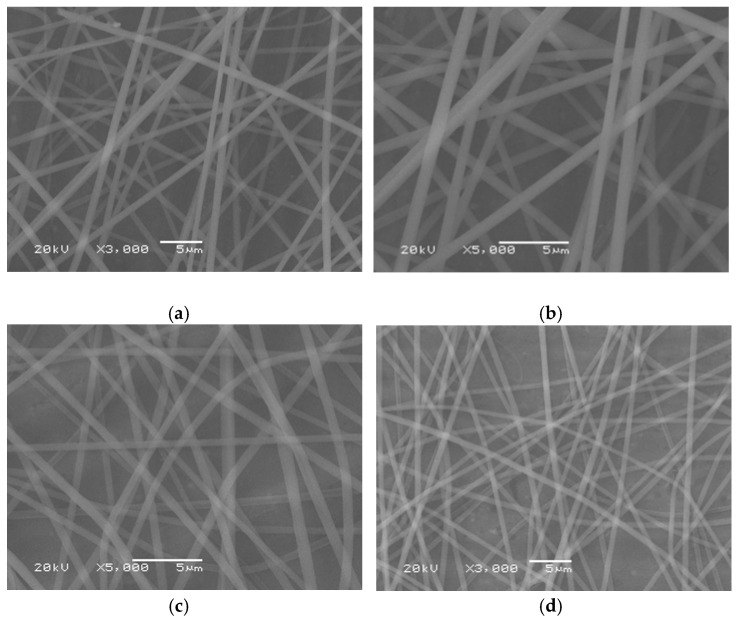
SEM images of the composite nanofibers of Eu(TFT)_3_phen/PMMA (**a**,**b**), Eu(TFT)_3_bpy/PMMA (**c**,**d**).

**Figure 4:**
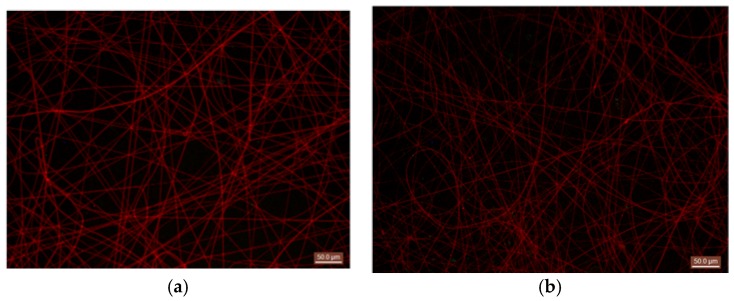
The fluorescence microscope images of Eu(TFT)_3_phen/PMMA (**a**) and Eu(TFT)_3_bpy/PMMA (**b**) composite nanofibers.

The SEM images of typical electrospun nanofibers demonstrated that the nanofibers of Eu(TFT)_3_phen/PMMA, Eu(TFT)_3_bpy/PMMA were successfully prepared (Figure 3a–d), and both nanofibers appeared to be distinctly separated and randomly interspersed. The surface of the composite nanofibers was smooth without identifiable particles, suggesting that complex **1** (or **2**) might be uniformly dispersed into the nanofibers. Figure 4a,b disposed the fluorescence microscope images of Eu(TFT)_3_phen/PMMA and Eu(TFT)_3_bpy/PMMA composite nanofibers, respectively. The carbonyl bond of PMMA and the rare earth ions could have produced coordination bonds. Their composite materials were used as electron transport carriers, and they could be fabricated as electrospinning fibers. More importantly, the aggregation of the Eu/PMMA composite led to the formation of nanoparticles in the composite fibers during the entire electrospinning process.

## 3. Results and Discussion

### 3.1. UV–Vis Spectral Analysis

**Figure 5:**
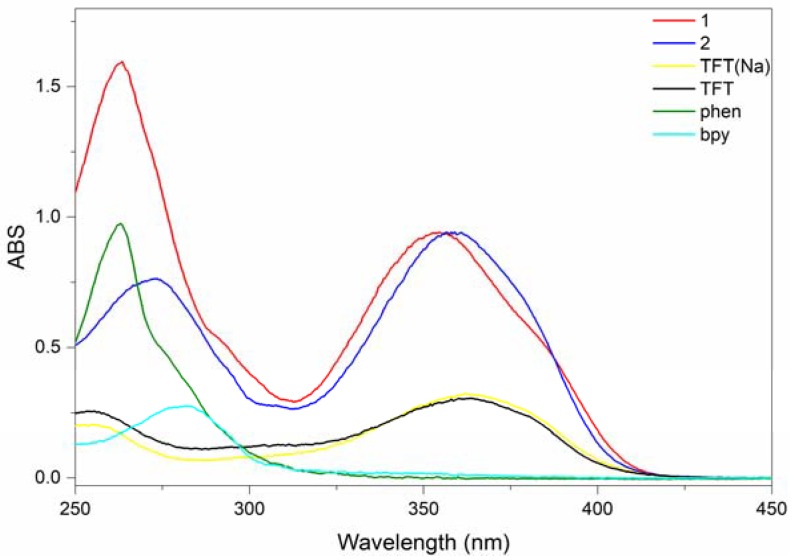
UV–Vis absorption spectra of complexes **1** and **2** and TFT, Na(TFT), phen, bpy in the CH_3_CN solution in the UV–Vis spectrum.

The ultraviolet absorption spectra of TFT and complexes **1** and **2** are shown in the CH_3_CN solution (1 × 10^−5^ mol·L^−1^). According to the spectra (Figure 5), the absorption broad band appeared to be slightly blue-shift from 363 nm for the TFT to 362 nm for the Na(TFT) because of the transfer of the β-diketonate proton. In addition, the maximal absorption of Phen and Bpy was 263 and 272 nm, respectively. This was due to the singlet–singlet π–π* absorption of the ancillary ligand. As for complexes **1** and **2**, the two typical UV–Vis absorption peaks were 353 and 359 nm, respectively. They had high absorption intensities, which were blue-shifted compared to ligand TFT (363 nm), due to the influence of TFT complexed with the Eu^3+^ ion. In addition, the molar absorption coefficient values of complexes **1** and **2** were approximately 0.9 × 10^5^ L·mol^−^^1^·cm^−1^ around 350–360 nm, which was three times higher than the ligand base on each of the complexes consisting of three ligands.

**Figure 6:**
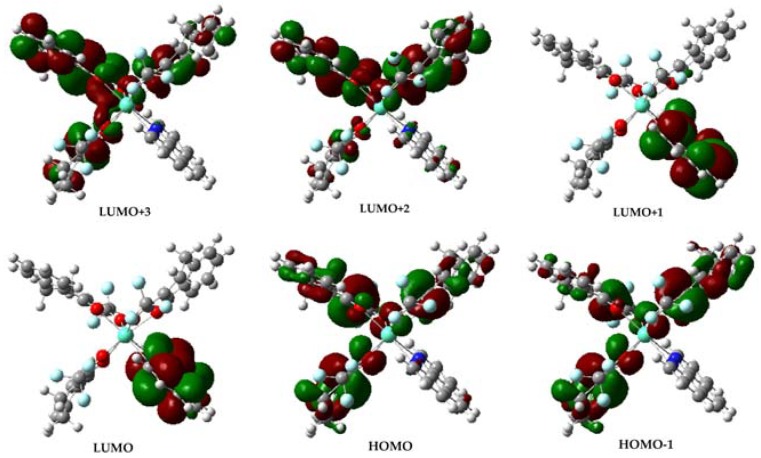
Spatial plots of the selected frontier molecular orbits of the excited states of complex **1**.

The calculated HOMO and LUMO levels of the ground state were −0.21 and −0.30 eV, respectively, while the values for singlet excitation of complex **1** were −0.08 and 0.05 eV (Table A3), respectively. Both the electronic distributions of HOMO localize at TFT, while those of LUMO localize at phen (Figure 6). The lowest excitation energy of complex **1** was 4.47 eV. The intraligand charge transfer, the metal-to-ligand charge transfer, and the metal center conversion are the major reasons for the absorption transitions. The calculation data corresponded with the phenomena that the absorption intensity of the complexes was approximately two times higher than that of the ligand.

### 3.2. Thermal Stability Analysis

**Figure 7:**
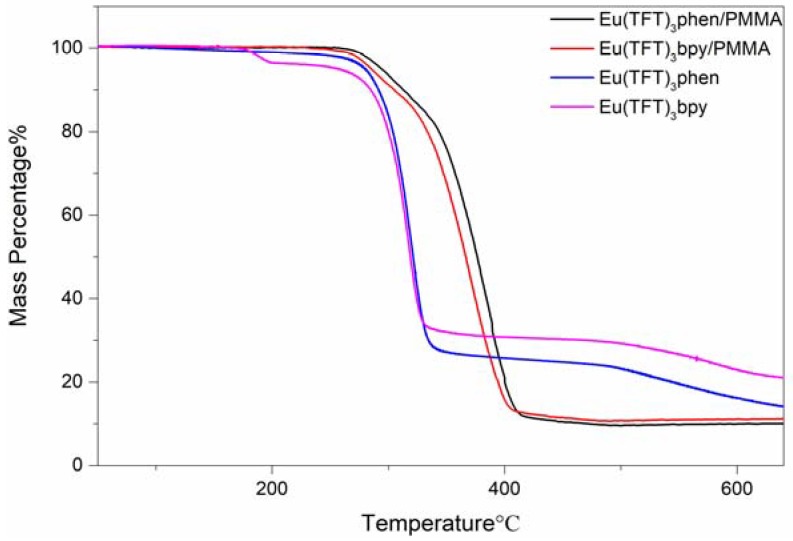
TGA curves of the complexes **1** and **2**, Eu(TFT)_3_phen/PMMA, and Eu(TFT)_3_bpy/PMMA.

The thermal stabilities of complexes **1** and **2** (Figure 7) were analyzed with thermogravimetric analysis (TGA) in a temperature range from 50 to 720 °C at a rate of 10 °C·min^−1^ under a nitrogenous gas atmosphere. Within the temperature range of 250–650 °C, the TGA curve of complex **1** showed that the weightlessness rate was 85.9%, which corresponds to how the TFT and phen ligands (calcd 86.1%) decomposed gradually. Within the temperature range of 190–210 °C, the TGA curve of complex **2** showed a weight loss of 4%, corresponding to how the solvent *n*-hexane (calcd 4%) evaporated gradually. In the temperature range of 250–650 °C, a weight loss of 76.2% corresponded to how the TFT and bpy ligands (calcd 76.8%) decomposed gradually. The final mass residues of complexes **1** and **2** were europium oxide.

We take complex **1** as an example. The experimental results show that the initial decomposition temperature of the composite is approximately 268 °C. It is higher than the decomposition temperature of complex **1** by 18 °C. The Eu/PMMA composite nanofiber produces a thermal behavior in a wide temperature range from 268 to 410 °C, which is 80°C higher than that of the undoped polymethyl methacrylate (PMMA) fiber. The result shows that when the PMMA is used as the polymer matrix, the rigid chain segment of the polymer has a limited effect on the vibration of the organic ligand and improves the relative independence of the doping molecule, such that the complex can remain stable in a good environment.

### 3.3. The Photoluminescent Properties of Complexes

**Figure 8:**
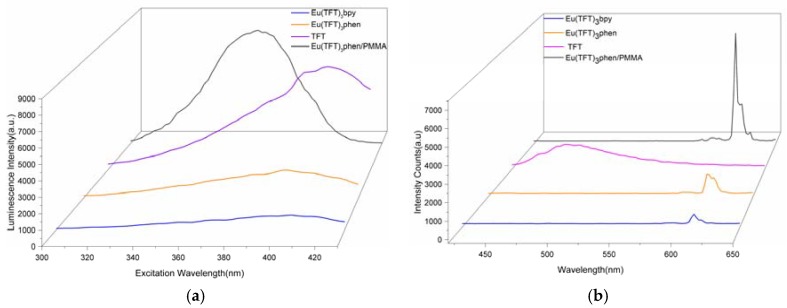
The excitation (**a**) and emission (**b**) spectra of complexes **1**, **2**, TFT, Eu(TFT)_3_phen/PMMA (12%) in the solid state.

The fluorescence spectra of TFT and complexes **1** and **2** are shown in Figure 8. The several characteristic narrow emission bands of the Eu^3+^ ion are formed upon excitation at 390–410 nm (Figure 8a), corresponding to the transitions from the metal-centered ^5^D_0_ excited state to the ^7^F_J_ ground state (Figure 8b), respectively. Among them, the ^5^D_0_ → ^7^F_J_ transition around 611 nm is the highest intensity emission, which belongs to an induced electric dipole transition, indicating that the Eu^3+^ ion is not situated in a location with inversion center symmetry.

The Eu(III) complexes have the strongest fluorescence intensity among the lanthanide series and emit red light at 605–700 nm. The blue light emission appeared at 450–480 nm. As can be seen in Figure 8b, the broad emission band of organic ligands (λ_em_ = 468 nm) cannot be observed in field of complexes **1** and **2**. Therefore, it is effective to transfer the absorbed energy to the emission level of the Eu^3+^ ion. Meanwhile, we discovered that the emission peak of complex **1** was stronger than complex **2**; the main reason for this was the difference in the auxiliary ligand. The light absorption of the auxiliary ligand of complexes **1** and **2** was different. The phen ligand had a higher efficiency of light absorption and an energy in the triplet excited state that was higher than the lowest emitting level of the Eu(III).

### 3.4. The Photoluminescent Properties of PMMA, PVP/Complexes Composites

**Figure 9:**
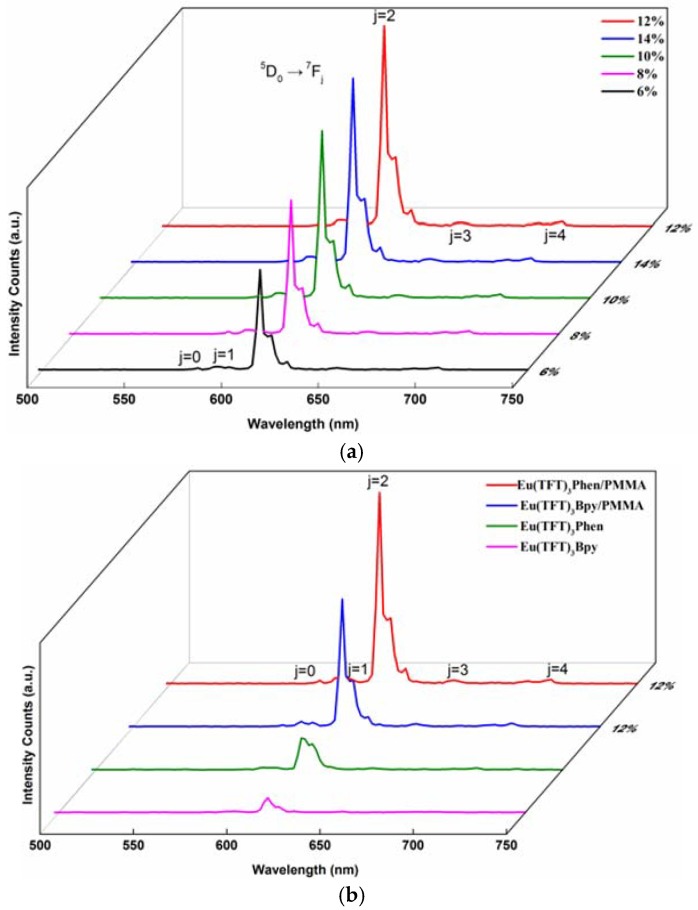
The emission spectra of the composite nanofibers Eu(TFT)_3_phen/PMMA (**a**); The emission spectra of complexes **1**, **2** and Eu/PVP composite fibers content of 12 wt % (**b**).

The emission spectra of the composite nanofibers with Eu(TFT)_3_phen/PMMA and the emission spectra of complexes **1** and **2** and Eu/PVP composite fibers with a content of 12 wt % are shown in Figure 9a,b. We found that the fluorescence intensity of the complexes in PMMA was significantly improved. With an increase in doping level, the fluorescence emission intensity increased gradually, then showed a decreasing trend after reaching a maximum strength with a Eu(TFT)_3_phen content of 12 wt % during emission. The ^5^D_0_ → ^7^F_2_ fluorescence emission peaks of Eu/PMMA composite fibers were higher than that of the Eu^3+^ complexes. The emission intensity of Eu(TFT)_3_phen/PMMA was clearly stronger than Eu(TFT)_3_bpy/PMMA, which is consistent with the fluorescence microscope images.

**Scheme 5:**
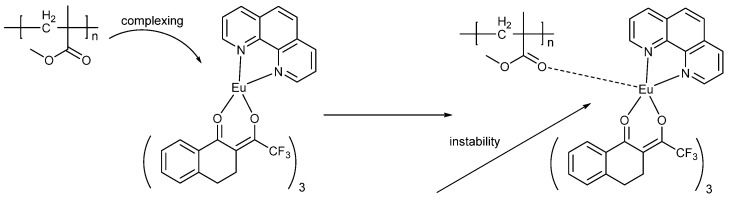
Coordination mechanism of Eu(TFT)_3_(phen)/PMMA.

**Figure 10:**
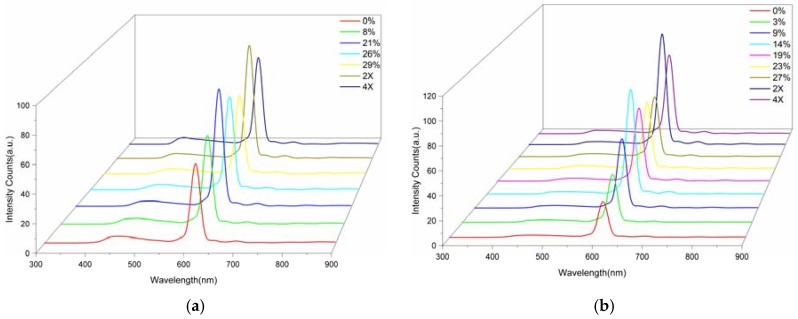
The liquid fluorescence curve spectrograms of complexes **3** (**a**) and **4** (**b**) in chloroform**.**

The polymers have a strong isolation effect, and they are not easy to react with small molecular compounds. By changing the mixed proportion of PMMA and complexes and measuring their fluorescence intensity, it was found that PMMA’s carbonyl bond may have coordinated with the rare earth ions to enhance the luminescence intensity of the rare earth ions (Scheme 5). In order to study the coordination and luminescence mechanism of the rare earth complexes and the polymers, we designed a series of experiments. Eu(TFT)_3_(TPPO) (0.07 g) and Eu(TFT)_3_(TPPO)_2_ (0.07 g) were dissolved in chloroform (50 mL), respectively. Then, polyvinyl pyrrolidone (PVP) was added to the solution to test their liquid fluorescence properties, and the liquid fluorescence spectra were obtained (Figure 10a,b). By analyzing the liquid fluorescence spectra, we found that the liquid fluorescence intensities of Eu(TFT)_3_(TPPO) and Eu(TFT)_3_(TPPO)_2_ were almost the same. This shows that the roles of two TPPO molecules and one TPPO molecule were almost identical. The fluorescence properties of the rare earth complexes depended on the electron-stretching transfer in the complexes. In the process of electron stretching in rare earth complexes, the difference in electronic tensile transmission between two TPPO molecules and one TPPO molecule was very small. However, when PVP was added, the liquid fluorescence curves changed significantly. The emission peak intensity was significantly improved at 612 nm. At approximately 500 nm, there was a strong launch peak, which was caused by PVP. PVP had an intramolecular electron transition. The carbonyl bond of PVP and the ligand coordinated, which could have enhanced the intramolecular transition of PVP and increased the aggregation-induction effect of molecules. The emission peaks of 500 and 612 nm exist simultaneously, which could have adjusted the luminescence color of the complexes (Figure 10a,b). When the mass fraction of Eu(TFT)_3_(TPPO)/PVP was 23 wt %, its liquid fluorescence intensity was at its highest (345 nm). When the mass fraction of Eu(TFT)_3_(TPPO)_2_/PVP was 15 wt %, its liquid fluorescence intensity was at its highest (345 nm). When the concentration was too high, they produced the aggregation-induced quenching, and the fluorescence intensity decreased. At the same time, the emission wavelengths of 500 and 612 nm were not quenched synchronously, indicating that the polymers were different from the fluorescence mechanism of the rare earth complexes. In theory, the rare earth ions and ligands could form twelve -coordination complexes, but because of space resistance, the trivalent rare earth ions and ligands could form stable complexes of eight-coordination, and the rare earth complexes of nine(ten)-coordination were not very stable.

The fluorescence intensities of the rare earth complexes were increased in methanol, 1,2-dimethoxyethane, and acetonitrile due to their association with the solvent. The oxygen atoms (nitrogen atoms) of the solvent and the Eu^3+^ ions of the rare earth complexes could form coordination bonds, which enhanced the fluorescence properties of the Eu(III) complexes. However, the coordination bond was unstable. The Eu(III) complexes could be mixed with PMMA, PVP, and polyacrylonitrile (PAN) to make composite nanofibers. The fluorescence properties of the mixed Eu(III) complexes with these polymers were improved significantly because these polymers and the Ln(III) ions of complexes could form new complexes. The different types of auxiliary ligands were added and the fluorescence intensity of the complexes could be clearly improved.

**Figure A13:**
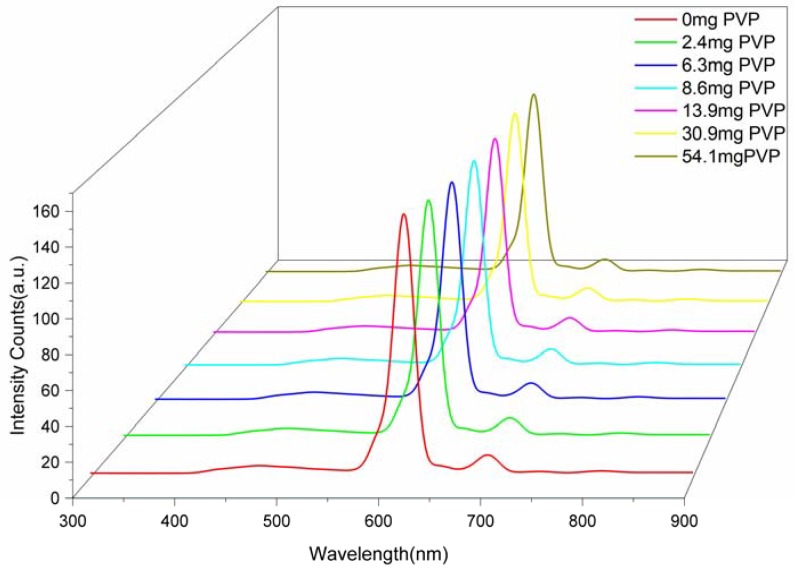
Fluorescence spectrum of PVP/complex **3** in dimethyl formamide (DMF).

Complex **3** (0.07 g) was added to *N*,*N*-dimethyl formamide (DMF) (50 mL). Then, PVP was added to the DMF solution. We found that the fluorescence intensity of the solution decreased slightly. The carbonyl bond of DMF could form a coordination bond with the Eu(III) ion of the rare earth complexes. However, the carbonyl bond of PVP, which is soluble in DMF, could not be coordinated with the complexes. There was no emission peak caused by PVP (Appendix C Figure A13). The emission peak near 500 nm was caused by the coordination of PVP and the rare earth complexes, which greatly enhanced the electron transition in polymer molecules. We found that the thermal stabilities of Eu(TFT)_3_phen/PMMA and Eu(TFT)_3_bpy/PMMA were improved by the analysis of heat loss. The experimental results showed that the oxygen atoms of PMMA and Eu(III) ions of complexes **1** and **2** formed new coordination bonds. Their thermal stability was enhanced and the fluorescence intensity in chloroform was significantly improved (Figure 10a,b). The fluorescence spectra indicated that the Eu/PMMA nanofibers had a maximum luminescence intensity at a content of 12 wt %. When PMMA had a mass fraction of 10–14 wt %, a carbonyl bond of PMMA was matched with an Eu(III) ion of complex **1** or **2**. Through a comparison with the fluorescence properties of the complexes **3** and **4** and Eu(TFT)_3_(TPPO)/PVP and Eu(TFT)_3_(TPPO)_2_/PVP, it was found that the rare earth complexes could form nine coordination bonds, which had a significant effect on the luminescence properties of the complexes. However, the ninth coordination bond was not a very stable coordination bond. The auxiliary ligands and the rare earth complexes of a different molar ratio formed new complexes, but their luminescence properties underwent little change. However, two different auxiliary ligands were involved in coordination, and the optical properties of the complexes could be greatly changed. The auxiliary ligands could be TPPO, phen, bpy, or the polymers with carbonyl groups.

**Figure 11:**
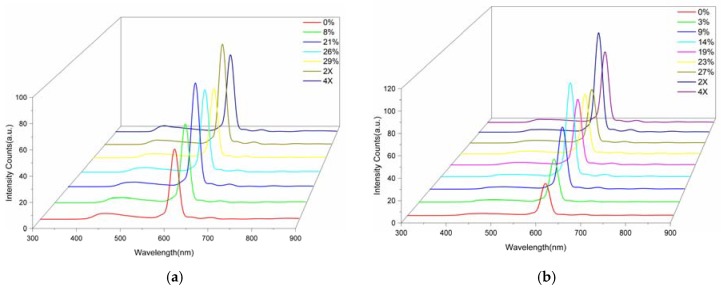
The liquid fluorescence curve spectrograms of complexes **5** (**a**) and **6** (**b**) in chloroform.

Complexes **5** and **6** were added to chloroform (50 mL), respectively. Then, PVP was added. The fluorescence intensity of PVP/complexes **5** and **6** were increased by one to three times, but it was far less than the fluorescence intensity of the PVP/complexes **3** and **4** (Figure 11a,b). The increase in the number of fluorine atoms in PFP would have enhanced the aggregation-induced effect of the rare earth complexes. From the experimental data, we found that when the concentration of PVP/complexes was diluted two times, the fluorescence intensity was significantly enhanced, which was due to the attenuation of the aggregation-induction effect. When the solution was diluted four times, the fluorescence intensity decreased due to a decrease in concentration.

### 3.5. Fluorescence Lifetimes and Fluorescence Quantum Efficiencies of Complexes ***1**–**6*** and Eu(TFT)_3_bpy/PMMA, Eu(TFT)_3_phen/PMMA

When the fluorescence lifetimes of complexes **1**–**6** and Eu(bpy)_3_bpy/PMMA, Eu(phen)_3_phen/PMMA were measured, it was found that the excited state molecules emitted fluorescence in the form of radiative transition to the ground state. However, there was also quenching and energy transfer between the molecules. The interaction between the molecules could accelerate the molecules of excited states back to the ground state, so the fluorescence lifetimes were reduced. The fluorescence lifetimes of the polymers were about 10^−8^ s, which was due to the strong aggregation-induced effect between the polymer molecules. The lifetime τ was related to the rate constant of these processes, and the rate constant k of the total withdrawal process could be expressed by the sum of the rate constants of various withdrawal processes (*k* = *k*_F_ + ∑*k*_i_ (*k*_F_: decay rate constant of various non-radiation processes); τ = 1/*k* = 1/(*k*_F_ + ∑*k*_i_)).

**Figure 12:**
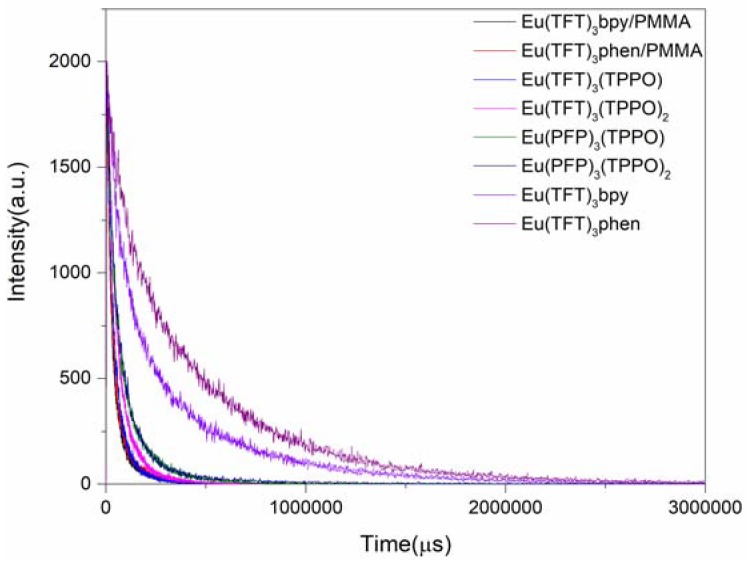
The fluorescence decay curves of complexes **1**–**6** and Eu(TFT)_3_bpy/PMMA, Eu(TFT)_3_phen/PVP.

When the rare earth complexes and the polymers were mixed, the fluorescence lifetime of the complex was reduced by coordinating with the polymer ligand with a high molecular weight, which consumed excessive energy. The fluorescence decay curves (Figure 12) and emission spectrums (Figure 8) of the rare earth complexes and Eu(TFT)_3_bpy/PMMA, Eu(TFT)_3_phen/PMMA showed that the coordination of PMMA and Eu(III) ions could increase the emission intensity of Eu(III) ions and increase the interaction between molecules.

The fluorescence quantum efficiencies of Eu(TFT)_3_phen/PMMA and Eu(TFT)_3_bpy/PMMA were attenuated compared to Eu(TFT)_3_phen and Eu(TFT)_3_bpy (Table A4). This is the same mechanism as the attenuation of the fluorescence lifetime of Eu(TFT)_3_phen/PMMA, which was mainly caused by the aggregation-induced quenching of the rare earth complexes and the polymers. However, by comparing the fluorescence lifetimes and quantum efficiencies of complexes **3**–**6**, the fluorescence lifetimes and quantum efficiencies of the rare earth complexes could be significantly improved with an increase in the number of auxiliary ligands. This is mainly because the two TPPO molecules were better for the sensitization of the rare earth ions.

## 4. Conclusions

In summary, we reported the preparation of six novel eight-coordinated Eu(III) complexes. Luminescence studies revealed that TFT, PFP, PMMA, and PVP were effective sensitizers on the luminescence of Eu(III) ions due to the ligand transferring the absorbed energy effectively to the emitting level of the center metal ion. In addition, the coordination and luminescence mechanism of the rare earth complexes and the polymers were investigated. The experimental data and results showed that the polymers could be used as an auxiliary ligand to change the optical properties of the rare earth complexes. The rare earth complexes could enhance electron transition in polymer molecules. The polymers and the complexes had two strong emission peaks, such that the light color was changed. A fluorine atom could enhance the aggregation-induced effect of PMMA and PVP/complexes.

## Figures and Tables

**Figure 1:**
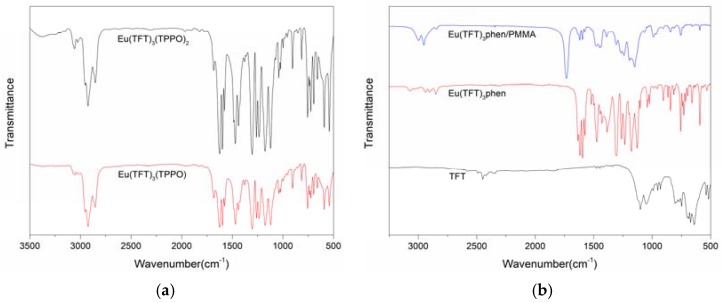
FTIR spectra of TFT, Eu(TFT)_3_(phen)/polymethyl methacrylate (PMMA) and complexes **2**–**4**.

**Figure A3:**
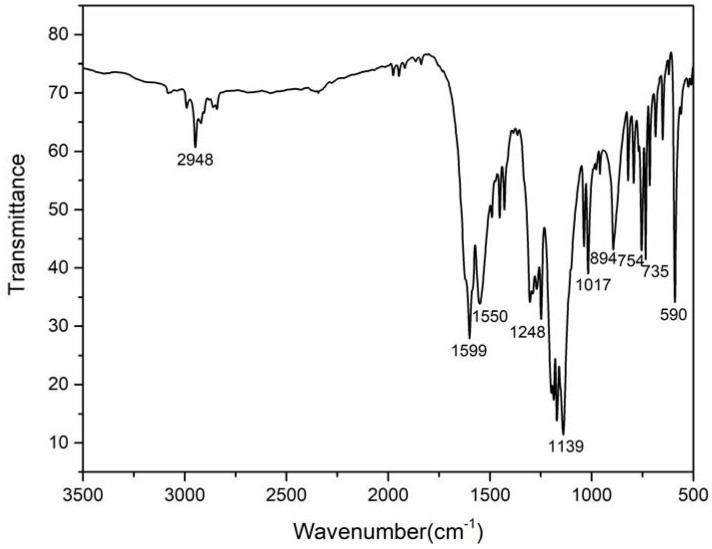
FTIR spectra of TFT.

**Figure A4:**
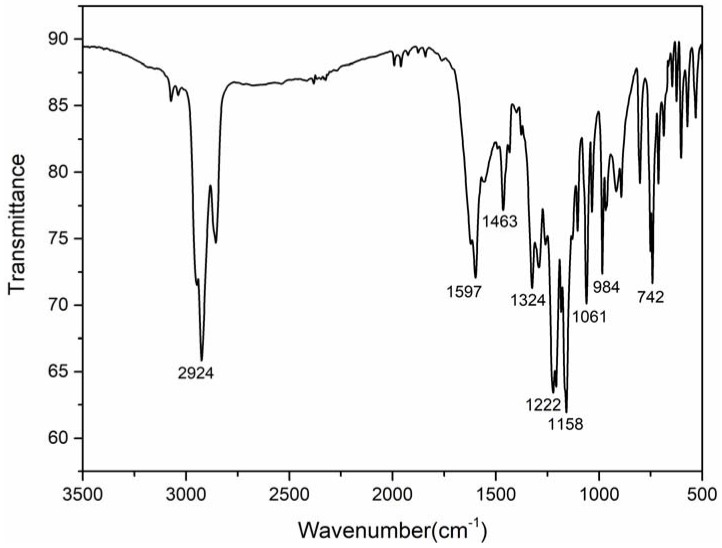
FTIR spectra of PFP.

**Figure A5:**
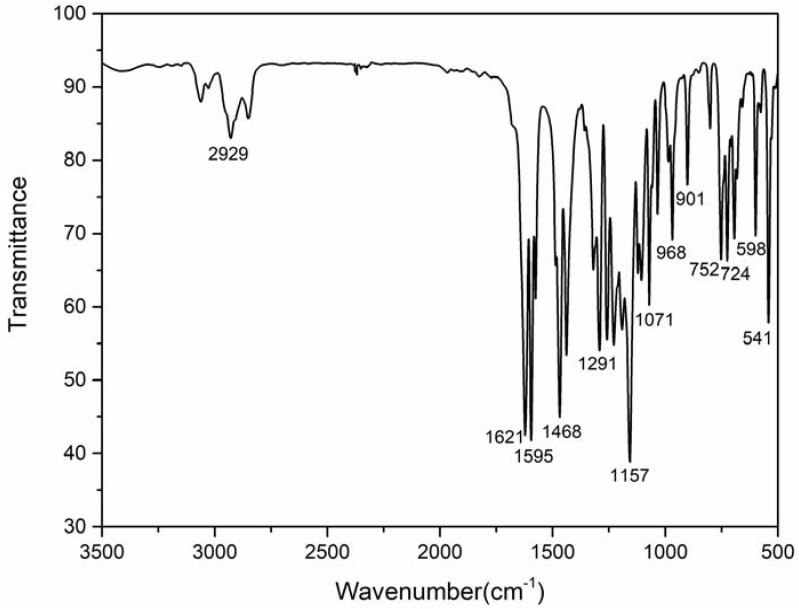
FTIR spectra of Eu(PFP)_3_TPPO.

**Figure A6:**
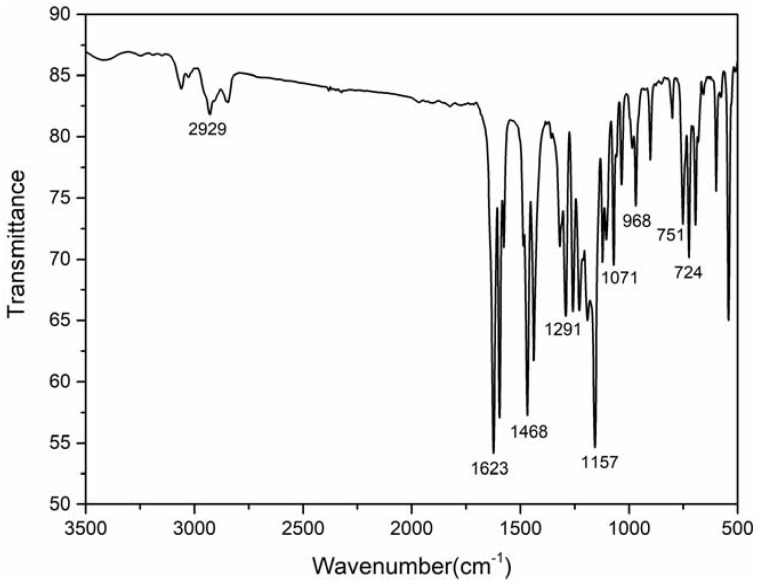
FTIR spectra of Eu(PFP)_3_(TPPO)_2_.

**Figure A7:**
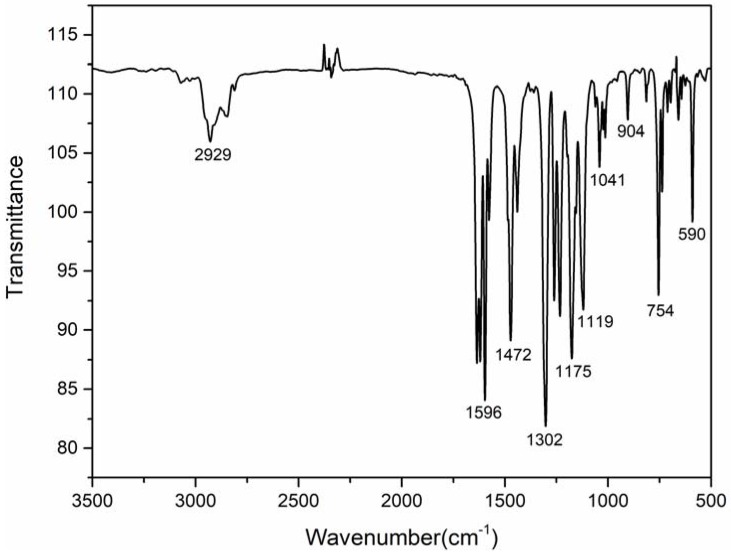
FTIR spectra of Eu(TFT)_3_bpy.

**Figure A8:**
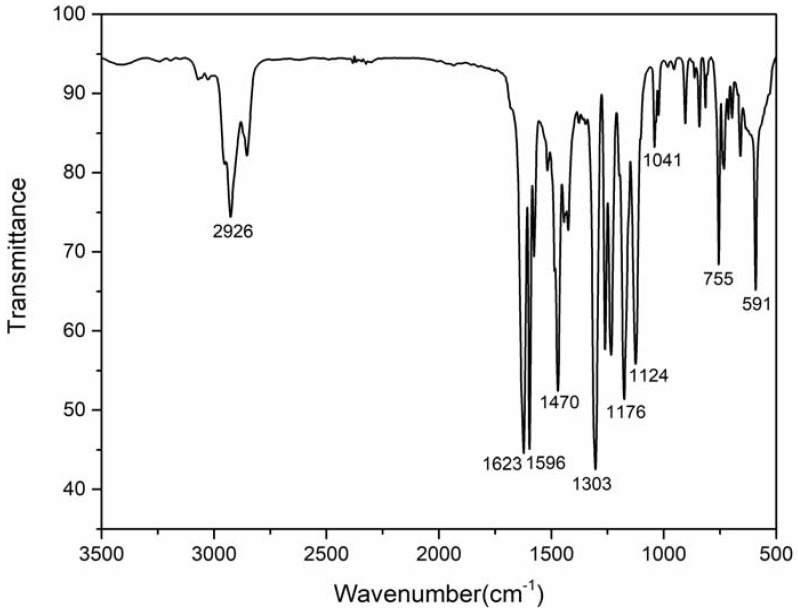
FTIR spectra of Eu(TFT)_3_phen.

**Figure A9:**
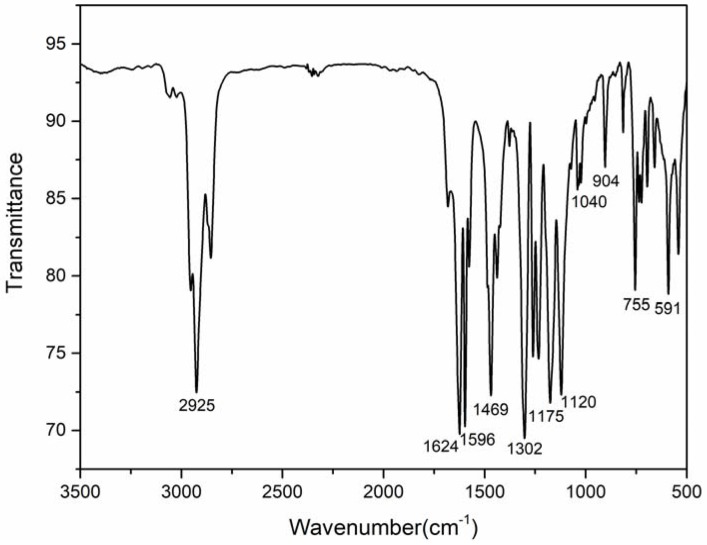
FTIR spectra of Eu(TFT)_3_TPPO.

**Figure A10:**
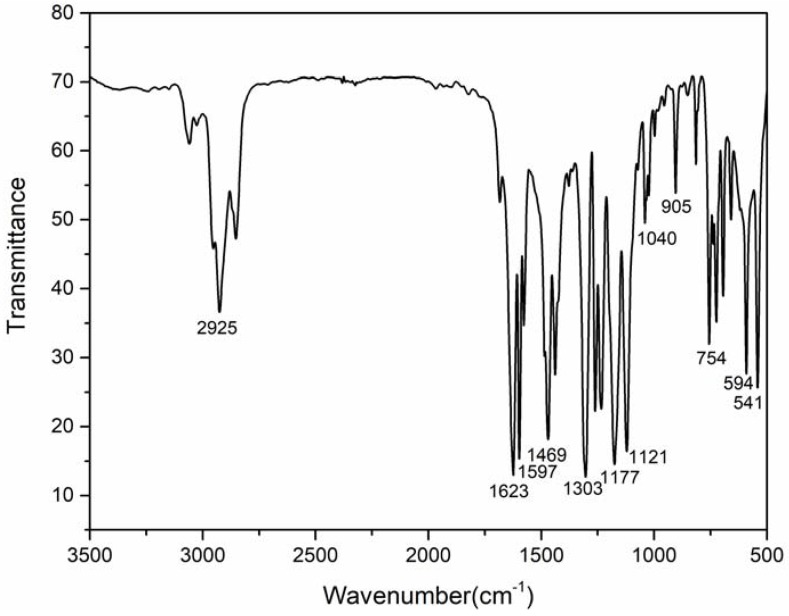
FTIR spectra of Eu(TFT)_3_(TPPO)_2_.

**Figure A11:**
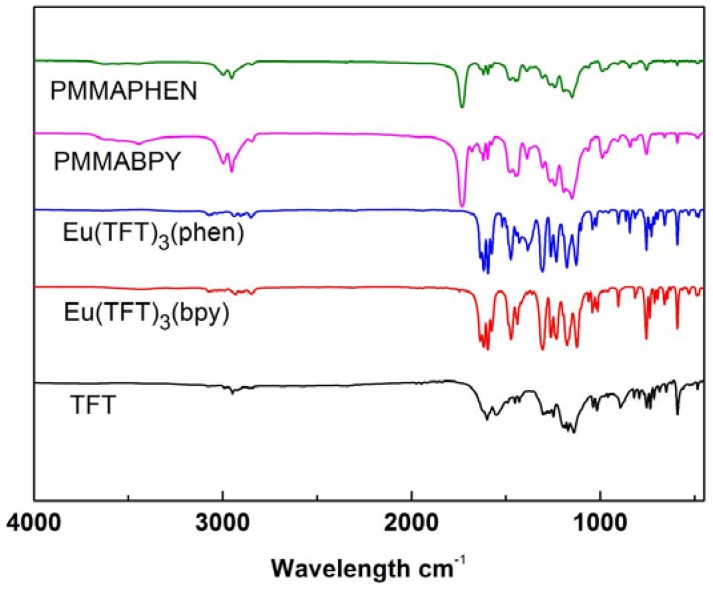
FTIR spectra of TFT, complexes **1** and **2**, and Eu/PMMA.

**Figure A12:**
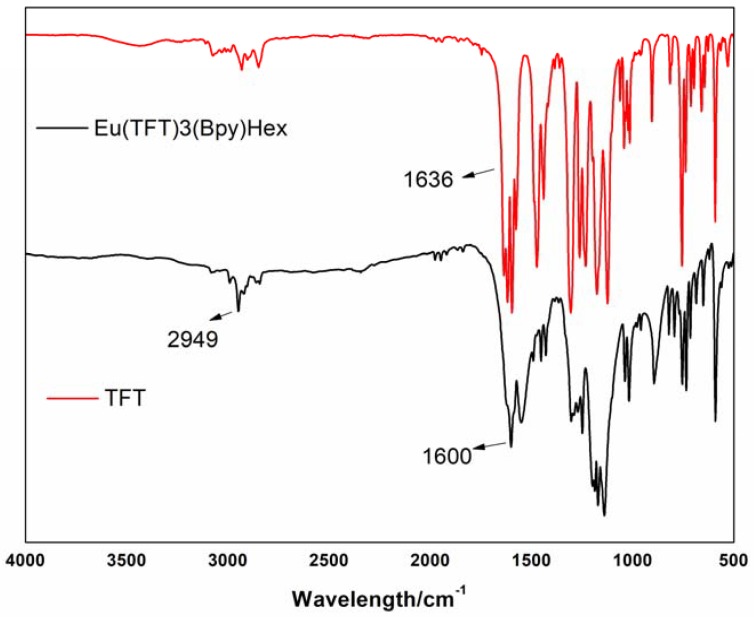
FTIR spectra of TFT and complex **2**.

## Supplementary Materials

The following are available online at http://www.mdpi.com/2073-4360/10/5/508/s1, Cif S1: CCDC 1484014-Complex **1**, Cif S2: CCDC 1524434-Complex **2**.

## Appendix D

**Table A1:** Selected bond lengths (Å) in Eu(TFT)_3_(phen).

Bond Lengths	
Eu(1)-O1	2.347(4)
Eu(1)-O2	2.351(5)
Eu(1)-O3	2.324(4)
Eu(1)-O4	2.347(4)
Eu(1)-O5	2.354(4)
Eu(1)-O6	2.358(4)
Eu(1)-N1	2.590(5)
Eu(1)-N2	2.586(5)

**Table A2:** Selected bond lengths (Å) in Eu(TFT)_3_(bpy)·Hex.

Bond Lengths			
Eu(1)-O1	2.341(3)	C(3S)-C(2S)	1.41(3)
Eu(1)-O2	2.368(3)	C(3S)-C(3S)#1	1.46(4)
Eu(1)-O3	2.356(3)	C(1S)-C(2S)	1.542(9)
Eu(1)-O4	2.347(3)		
Eu(1)-O5	2.351(3)		
Eu(1)-O6	2.379(3)		
Eu(1)-N1	2.576(3)		
Eu(1)-N2	2.590(8)		

**Table A3:** Absorptions of Eu complex 1 Calculated with the TDDFT Method.

Sn	Confign	CI codff	E/nm (eV)	Oscillator	Assignt
S1	H → L + 2	0.2898	277.23(4.47)	0.1533	ILCT/LMCT/MLCT/MC
S2	H → L + 3	0.3316	275.69(4.49)	0.3093	ILCT
S3	H-1 → L + 2	0.1572	266.16(4.66)	0.4822	ILCT/LMCT/MLCT/MC
S4	H → L + 1	0.1091	254.70(4.87)	0.0279	LMCT/MLCT/MC
S5	H → L	0.1363	249.71(4.97)	0.0024	LMCT/MLCT/MC

**Table A4:** Luminescence lifetimes and quantum yields of complexes.

Samples	τ (ms)	Ф_overall_ (%)
Eu(TFT)_3_phen	314	52.17
Eu(TFT)_3_bpy	172	48.93
Eu(TFT)_3_phen/PMMA	28	19.17
Eu(TFT)_3_bpy/PMMA	26	18.81
Eu(TFT)_3_(TPPO)	45.2	20.76
Eu(TFT)_3_(TPPO)_2_	62.4	21.97
Eu(PFP)_3_(TPPO)	62	21.91
Eu(PFP)_3_(TPPO)_2_	70	22.17
