# Supplementary material for: The Coordination and Luminescence of the Eu(III) Complexes with the Polymers (PMMA, PVP)
Source: Polymers (Basel). 2018 May 7;10(5):508. doi: 10.3390/polym10050508 (PMC6415368; doi:10.3390/polym10050508)

checkCIF/PLATON report

Structure factors have been supplied for datablock(s) HDS161202_0m

THIS REPORT IS FOR GUIDANCE ONLY. IF USED AS PART OF A REVIEW PROCEDURE FOR PUBLICATION, IT SHOULD NOT REPLACE THE EXPERTISE OF AN EXPERIENCED CRYSTALLOGRAPHIC REFEREE.

No syntax errors found. CIF dictionary Interpreting this report

Datablock: HDS161202_0m

```
Bond precision:  C-C = 0.0093 A          Wavelength=0.71073

Cell:           a=12.0336(16)    b=21.134(3)        c=19.0963(17)
                alpha=90         beta=110.757(6)    gamma=90
Temperature:    296 K
```

	Calculated	Reported
Volume	4541.3(10)	4541.3(10)
Space group	P 21/c	P 21/c
Hall group	-P 2ybc	-P 2ybc
Moiety formula	C46 H32 Eu F9 N2 O6, 0.5(C6 H14)	C46 H32 Eu F9 N2 O6, 0.5(C6H14)
Sum formula	C49 H39 Eu F9 N2 O6	C49 H39 Eu F9 N2 O6
Mr	1074.79	1074.78
Dx,g cm-3	1.572	1.572
Z	4	4
Mu (mm-1)	1.470	1.470
F000	2156.0	2156.0
F000’	2156.64	
h,k,lmax	15,28,25	15,28,24
Nref	11141	11028
Tmin,Tmax	0.669,0.768	0.960,1.000
Tmin’	0.656	

```
Correction method= # Reported T Limits: Tmin=0.960 Tmax=1.000
AbsCorr = MULTI-SCAN

Data completeness= 0.990          Theta(max)= 28.164

R(reflections)= 0.0430( 7033)     wR2(reflections)= 0.1134( 11028)

S = 1.066                  Npar= 605
```

The following ALERTS were generated. Each ALERT has the format
test-name_ALERT_alert-type_alert-level.
Click on the hyperlinks for more details of the test.

Alert level C

```
PLAT230_ALERT_2_C Hirshfeld Test Diff for    F6     --  C24     ..        6.5 s.u.
PLAT230_ALERT_2_C Hirshfeld Test Diff for    C25    --  C30     ..        5.2 s.u.
PLAT234_ALERT_4_C Large Hirshfeld Difference C3     --  C4      ..       0.16 Ang.
PLAT234_ALERT_4_C Large Hirshfeld Difference C5     --  C10     ..       0.16 Ang.
PLAT241_ALERT_2_C High   'MainMol' Ueq as Compared to Neighbors of        C10 Check
PLAT241_ALERT_2_C High   'MainMol' Ueq as Compared to Neighbors of        C27 Check
PLAT241_ALERT_2_C High   'MainMol' Ueq as Compared to Neighbors of        C34 Check
PLAT244_ALERT_4_C Low    'Solvent' Ueq as Compared to Neighbors of        C2S Check
PLAT342_ALERT_3_C Low Bond Precision on  C-C Bonds ...............    0.00927 Ang.
PLAT360_ALERT_2_C Short  C(sp3)-C(sp3) Bond   C2S    -   C3S    ..       1.41 Ang.
PLAT905_ALERT_3_C Negative K value in the Analysis of Variance ...     -1.140 Report
PLAT910_ALERT_3_C Missing # of FCF Reflection(s) Below Theta(Min)           5 Note
PLAT911_ALERT_3_C Missing # FCF Refl Between THmin & STh/L=  0.600         28 Report
```

Alert level G

```
PLAT002_ALERT_2_G Number of Distance or Angle Restraints on AtSite          5 Note
PLAT003_ALERT_2_G Number of Uiso or Uij Restrained non-H Atoms ...          4 Report
PLAT042_ALERT_1_G Calc. and Reported MoietyFormula Strings  Differ     Please Check
PLAT128_ALERT_4_G Alternate Setting for Input Space Group  P21/c        P21/n Note
PLAT172_ALERT_4_G The CIF-Embedded .res File Contains DFIX Records          3 Report
PLAT177_ALERT_4_G The CIF-Embedded .res File Contains DELU Records          2 Report
PLAT186_ALERT_4_G The CIF-Embedded .res File Contains ISOR Records          1 Report
PLAT242_ALERT_2_G Low    'MainMol' Ueq as Compared to Neighbors of        C12 Check
PLAT242_ALERT_2_G Low    'MainMol' Ueq as Compared to Neighbors of        C24 Check
PLAT242_ALERT_2_G Low    'MainMol' Ueq as Compared to Neighbors of        C36 Check
PLAT720_ALERT_4_G Number of Unusual/Non-Standard Labels ..........          7 Note
PLAT860_ALERT_3_G Number of Least-Squares Restraints .............         11 Note
PLAT912_ALERT_4_G Missing # of FCF Reflections Above STh/L=  0.600         66 Note
PLAT933_ALERT_2_G Number of OMIT Records in Embedded .res File ...          3 Note
PLAT978_ALERT_2_G Number C-C Bonds with Positive Residual Density.          1 Note
```

---

0 **ALERT level A** = Most likely a serious problem - resolve or explain
0 **ALERT level B** = A potentially serious problem, consider carefully
13 **ALERT level C** = Check. Ensure it is not caused by an omission or oversight
15 **ALERT level G** = General information/check it is not something unexpected

1 ALERT type 1 CIF construction/syntax error, inconsistent or missing data
13 ALERT type 2 Indicator that the structure model may be wrong or deficient
5 ALERT type 3 Indicator that the structure quality may be low
9 ALERT type 4 Improvement, methodology, query or suggestion
0 ALERT type 5 Informative message, check

It is advisable to attempt to resolve as many as possible of the alerts in all categories. Often the minor alerts point to easily fixed oversights, errors and omissions in your CIF or refinement strategy, so attention to these fine details can be worthwhile. In order to resolve some of the more serious problems it may be necessary to carry out additional measurements or structure refinements. However, the purpose of your study may justify the reported deviations and the more serious of these should normally be commented upon in the discussion or experimental section of a paper or in the "special_details" fields of the CIF. checkCIF was carefully designed to identify outliers and unusual parameters, but every test has its limitations and alerts that are not important in a particular case may appear. Conversely, the absence of alerts does not guarantee there are no aspects of the results needing attention. It is up to the individual to critically assess their own results and, if necessary, seek expert advice.

Publication of your CIF in IUCr journals

A basic structural check has been run on your CIF. These basic checks will be run on all CIFs submitted for publication in IUCr journals (*Acta Crystallographica*, *Journal of Applied Crystallography*, *Journal of Synchrotron Radiation*); however, if you intend to submit to *Acta Crystallographica Section C* or *E* or *IUCrData*, you should make sure that full publication checks are run on the final version of your CIF prior to submission.

Publication of your CIF in other journals

Please refer to the *Notes for Authors* of the relevant journal for any special instructions relating to CIF submission.

PLATON version of 27/03/2017; check.def file version of 24/03/2017

Datablock HDS161202_0m - ellipsoid plot

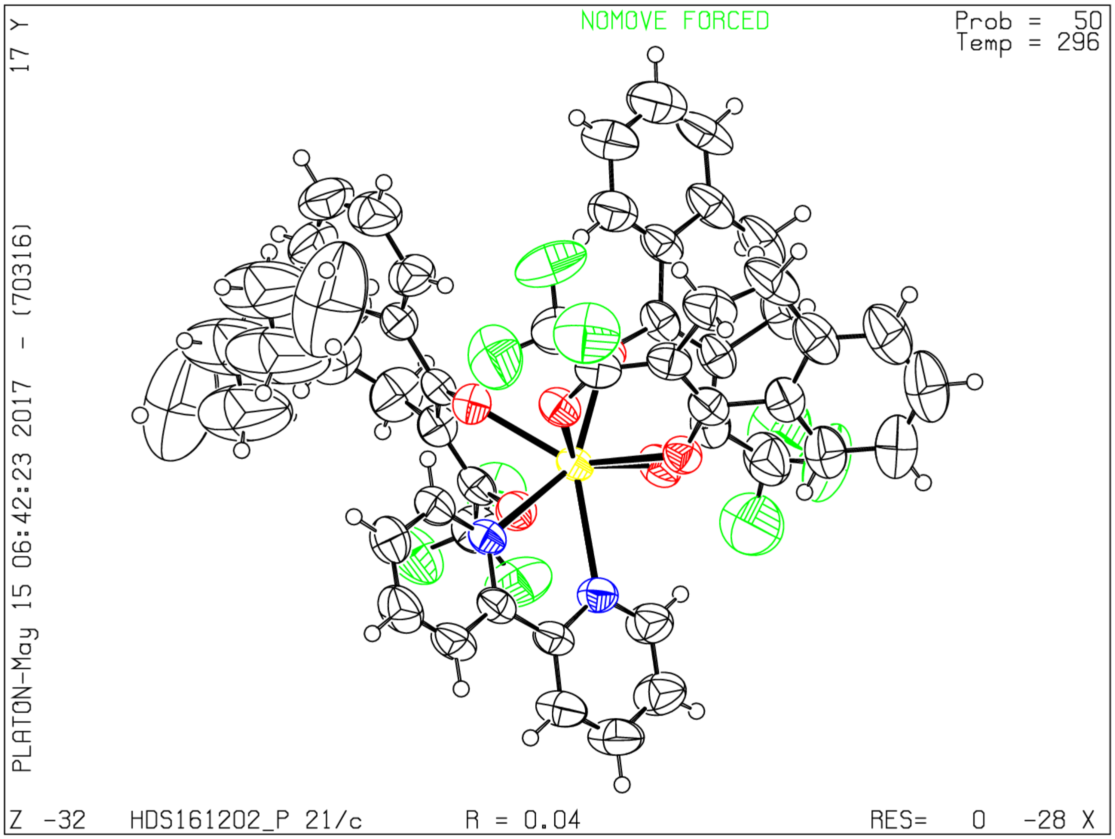

Supplement: Supplementary file 1 [file polymers-10-00508-s001.zip › checkcif for CCDC 1524434-Complex 2.pdf]
