# Supplementary material for: The Coordination and Luminescence of the Eu(III) Complexes with the Polymers (PMMA, PVP)
Source: Polymers (Basel). 2018 May 7;10(5):508. doi: 10.3390/polym10050508 (PMC6415368; doi:10.3390/polym10050508)

checkCIF/PLATON report

Structure factors have been supplied for datablock(s) HD61S5_0m

THIS REPORT IS FOR GUIDANCE ONLY. IF USED AS PART OF A REVIEW PROCEDURE FOR PUBLICATION, IT SHOULD NOT REPLACE THE EXPERTISE OF AN EXPERIENCED CRYSTALLOGRAPHIC REFEREE.

No syntax errors found.   CIF dictionary   Interpreting this report

Datablock: HD61S5_0m

```
Bond precision:  C-C = 0.0126 A          Wavelength=0.71073

Cell:            a=21.005(2)     b=9.6340(9)          c=22.989(2)
                 alpha=90        beta=105.155(1)      gamma=90
Temperature:     296 K

                Calculated                  Reported
Volume          4490.3(7)                   4490.3(7)
Space group     P 21/n                      P 21/n
Hall group      -P 2yn                      -P 2yn
Moiety formula  C48 H30 Eu F9 N2 O6         C48 H30 Eu F9 N2 O6
Sum formula     C48 H30 Eu F9 N2 O6         C48 H30 Eu F9 N2 O6
Mr              1053.71                     1053.70
Dx,g cm-3       1.559                       1.559
Z               4                           4
Mu (mm-1)       1.485                       1.485
F000            2096.0                      2096.0
F000'           2096.63
h,k,lmax        28,12,30                    28,12,30
Nref            11246                       11190
Tmin,Tmax       0.628,0.680                 0.994,1.000
Tmin'           0.616

Correction method= # Reported T Limits: Tmin=0.994 Tmax=1.000
AbsCorr = MULTI-SCAN

Data completeness= 0.995          Theta(max)= 28.384

R(reflections)= 0.0507( 5646)     wR2(reflections)= 0.1447( 11190)

S = 0.959                  Npar= 589
```

The following ALERTS were generated. Each ALERT has the format
test-name_ALERT_alert-type_alert-level.
Click on the hyperlinks for more details of the test.

Alert level C

```
PLAT234_ALERT_4_C Large Hirshfeld Difference F6     --  C36     ..       0.17 Ang.
PLAT234_ALERT_4_C Large Hirshfeld Difference F9     --  C48     ..       0.18 Ang.
PLAT234_ALERT_4_C Large Hirshfeld Difference C7     --  C8      ..       0.16 Ang.
PLAT234_ALERT_4_C Large Hirshfeld Difference C7     --  C10     ..       0.16 Ang.
PLAT234_ALERT_4_C Large Hirshfeld Difference C20    --  C21     ..       0.16 Ang.
PLAT234_ALERT_4_C Large Hirshfeld Difference C20    --  C23     ..       0.19 Ang.
PLAT234_ALERT_4_C Large Hirshfeld Difference C23    --  C24     ..       0.17 Ang.
PLAT234_ALERT_4_C Large Hirshfeld Difference C25    --  C30     ..       0.16 Ang.
PLAT234_ALERT_4_C Large Hirshfeld Difference C29    --  C34     ..       0.18 Ang.
PLAT241_ALERT_2_C High   'MainMol' Ueq as Compared to Neighbors of         C8 Check
PLAT241_ALERT_2_C High   'MainMol' Ueq as Compared to Neighbors of        C21 Check
PLAT241_ALERT_2_C High   'MainMol' Ueq as Compared to Neighbors of        C22 Check
PLAT241_ALERT_2_C High   'MainMol' Ueq as Compared to Neighbors of        C27 Check
PLAT241_ALERT_2_C High   'MainMol' Ueq as Compared to Neighbors of        C39 Check
PLAT241_ALERT_2_C High   'MainMol' Ueq as Compared to Neighbors of        C41 Check
PLAT241_ALERT_2_C High   'MainMol' Ueq as Compared to Neighbors of        C46 Check
PLAT242_ALERT_2_C Low    'MainMol' Ueq as Compared to Neighbors of        Eu1 Check
PLAT242_ALERT_2_C Low    'MainMol' Ueq as Compared to Neighbors of        C17 Check
PLAT242_ALERT_2_C Low    'MainMol' Ueq as Compared to Neighbors of        C23 Check
PLAT242_ALERT_2_C Low    'MainMol' Ueq as Compared to Neighbors of        C42 Check
PLAT242_ALERT_2_C Low    'MainMol' Ueq as Compared to Neighbors of        C44 Check
PLAT242_ALERT_2_C Low    'MainMol' Ueq as Compared to Neighbors of        C47 Check
PLAT342_ALERT_3_C Low Bond Precision on  C-C Bonds ...............    0.01261 Ang.
PLAT360_ALERT_2_C Short  C(sp3)-C(sp3) Bond   C45    -   C46    ..       1.37 Ang.
PLAT369_ALERT_2_C Long    C(sp2)-C(sp2) Bond  C20    -   C21    ..       1.53 Ang.
PLAT410_ALERT_2_C Short Intra H...H Contact   H45A   ..  H46B    .       1.99 Ang.
PLAT410_ALERT_2_C Short Intra H...H Contact   H45B   ..  H46A    .       1.98 Ang.
PLAT482_ALERT_4_C Small D-H..A Angle Rep for C13    ..  O1      ..      99.70 Degree
PLAT601_ALERT_2_C Structure Contains Solvent Accessible VOIDS of .         38 Ang3
PLAT906_ALERT_3_C Large K value in the Analysis of Variance ......      4.424 Check
```

Alert level G

```
PLAT002_ALERT_2_G Number of Distance or Angle Restraints on AtSite          2 Note
PLAT003_ALERT_2_G Number of Uiso or Uij Restrained non-H Atoms ...          9 Report
PLAT171_ALERT_4_G The CIF-Embedded .res File Contains EADP Records          1 Report
PLAT172_ALERT_4_G The CIF-Embedded .res File Contains DFIX Records          1 Report
PLAT186_ALERT_4_G The CIF-Embedded .res File Contains ISOR Records          2 Report
PLAT242_ALERT_2_G Low    'MainMol' Ueq as Compared to Neighbors of        C24 Check
PLAT242_ALERT_2_G Low    'MainMol' Ueq as Compared to Neighbors of        C36 Check
PLAT242_ALERT_2_G Low    'MainMol' Ueq as Compared to Neighbors of        C48 Check
PLAT343_ALERT_2_G Unusual sp3      Angle Range in Main Residue for        C46 Check
PLAT860_ALERT_3_G Number of Least-Squares Restraints .............         55 Note
PLAT910_ALERT_3_G Missing # of FCF Reflection(s) Below Theta(Min)           4 Note
PLAT912_ALERT_4_G Missing # of FCF Reflections Above STh/L=  0.600         53 Note
PLAT913_ALERT_3_G Missing # of Very Strong Reflections in FCF ....          1 Note
PLAT978_ALERT_2_G Number C-C Bonds with Positive Residual Density.          2 Note
```

---

0 **ALERT level A** = Most likely a serious problem - resolve or explain
0 **ALERT level B** = A potentially serious problem, consider carefully
30 **ALERT level C** = Check. Ensure it is not caused by an omission or oversight
14 **ALERT level G** = General information/check it is not something unexpected

0 ALERT type 1 CIF construction/syntax error, inconsistent or missing data
25 ALERT type 2 Indicator that the structure model may be wrong or deficient
5 ALERT type 3 Indicator that the structure quality may be low
14 ALERT type 4 Improvement, methodology, query or suggestion
0 ALERT type 5 Informative message, check

It is advisable to attempt to resolve as many as possible of the alerts in all categories. Often the minor alerts point to easily fixed oversights, errors and omissions in your CIF or refinement strategy, so attention to these fine details can be worthwhile. In order to resolve some of the more serious problems it may be necessary to carry out additional measurements or structure refinements. However, the purpose of your study may justify the reported deviations and the more serious of these should normally be commented upon in the discussion or experimental section of a paper or in the "special_details" fields of the CIF. checkCIF was carefully designed to identify outliers and unusual parameters, but every test has its limitations and alerts that are not important in a particular case may appear. Conversely, the absence of alerts does not guarantee there are no aspects of the results needing attention. It is up to the individual to critically assess their own results and, if necessary, seek expert advice.

Publication of your CIF in IUCr journals

A basic structural check has been run on your CIF. These basic checks will be run on all CIFs submitted for publication in IUCr journals (*Acta Crystallographica*, *Journal of Applied Crystallography*, *Journal of Synchrotron Radiation*); however, if you intend to submit to *Acta Crystallographica Section C* or *E* or *IUCrData*, you should make sure that full publication checks are run on the final version of your CIF prior to submission.

Publication of your CIF in other journals

Please refer to the *Notes for Authors* of the relevant journal for any special instructions relating to CIF submission.

PLATON version of 27/03/2017; check.def file version of 24/03/2017

Datablock HD61S5_0m - ellipsoid plot

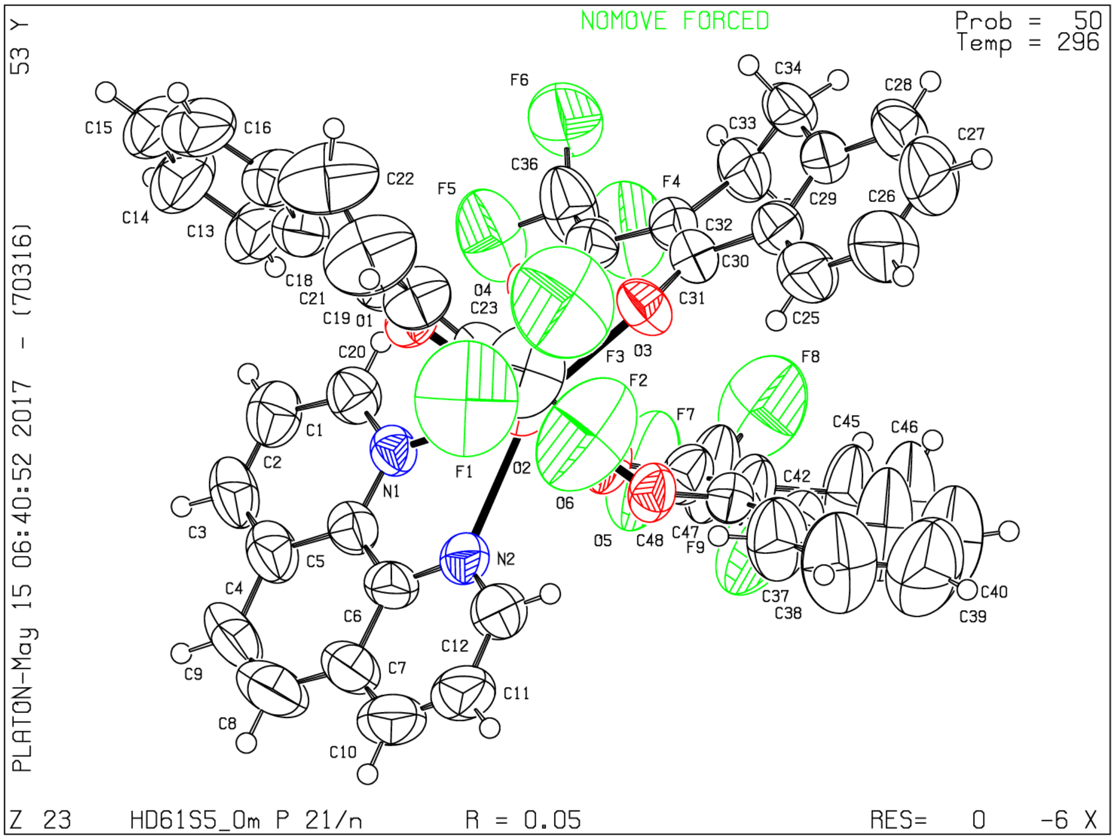

Supplement: Supplementary file 1 [file polymers-10-00508-s001.zip › checkcif for CCDC 1484014-Complex 1.pdf]
